# Role of endogenous and exogenous attention in task-relevant visual perceptual learning

**DOI:** 10.1371/journal.pone.0237912

**Published:** 2020-08-28

**Authors:** Kieu Ngoc Nguyen, Takeo Watanabe, George John Andersen

**Affiliations:** 1 Department of Psychology, University of California, Riverside, Riverside, California, United States of America; 2 Department of Cognitive, Linguistic, and Psychological Sciences, Brown University, Providence, Rhode Island, United States of America; University of Bath, UNITED KINGDOM

## Abstract

The present study examined the role of exogenous and endogenous attention in task relevant visual perceptual learning (TR-VPL). VPL performance was assessed by examining the learning to a trained stimulus feature and transfer of learning to an untrained stimulus feature. To assess the differential role of attention in VPL, two types of attentional cues were manipulated; exogenous and endogenous. In order to assess the effectiveness of the attentional cue, the two types of attentional cues were further divided into three cue-validity conditions. Participants were trained, on a novel task, to detect the presence of a complex gabor patch embedded in fixed Gaussian contrast noise while contrast thresholds were varied. The results showed initial differences were found prior to training, and so the magnitude of learning was assessed. Exogenous and endogenous attention were both found to facilitate learning and feature transfer when investigating pre-test and post-test thresholds. However, examination of training data indicate attentional differences; with endogenous attention showing consistently lower contrast thresholds as compared to exogenous attention suggesting greater impact of training with endogenous attention. We conclude that several factors, including the use of stimuli that resulted in rapid learning, may have contributed to the generalization of learning found in the present study.

## Introduction

Through repeated exposure or training of visual stimuli, improvements in performance can result in enhanced visual processing known as visual perceptual learning (VPL). These perceptual improvements have been found to be long-lasting [1–3] and can result in changes in neural processing, known as neural plasticity [4, 5]. VPL research has been focused not only on understanding learning in visual processing but also identifying fundamental principles common to learning in general. In addition, many aspects of early visual processing decline with normal aging. These declines include decreased performance in spatial vision, contrast sensitivity, orientation, and motion—all of which are important for higher-level visual tasks such as driving [6]. Additionally, those suffering from visual deficits, such as macular degeneration [7], presbyopia [8, 9], or amblyopia [10–13], might benefit from VPL protocols. Thus, elucidating the mechanisms that underlie learning has important implications for the development and application of VPL in clinical and practical settings.

Research on VPL has revealed a diverse set of findings that have resulted in different theories of VPL. These findings have centered on whether aspects of VPL are specific or generalizable [14–17] as well as the changes in neural visual processing due to VPL [18–20]. Specificity is the failure for training-induced performance improvements to transfer, or generalize, to an untrained stimuli or task. VPL has been found to be dependent on many factors including the learned visual feature, the type of task, and exposure to a feature without a task [21]. Performance improvements from training have been found to be specific to early level attributes of a stimulus ranging from orientation [22, 23] and spatial frequency [22, 24] to motion [25, 26] and contrast [27–29] (for detailed reviews of the extent of specificity in PL see [30] or [31]). This specificity has been taken to indicate that changes in processing occur at early cortical sites where primitive features are processed. Variations of the training task such as, task difficulty [14], high-precision stimuli [16], or long training sessions [32] could lead to some degree of learning specificity. VPL was reported to transfer across task [33], stimulus features [34], and retinal location [35] under different training conditions. This generalization suggests that the locus of learning might be at higher-level processing regions in which changes in reweighting of read-out connections or the level at which response decisions occur [36–40]. Generalization of VPL has also been found to occur for tasks that employ easy training [14, 41], shorter training sessions [32], shorter training trials [42], or double-training [35].

Previous research on VPL has found two different types of VPL; task relevant VPL (TR-VPL) and task-irrelevant VPL (TI-VPL). TR-VPL is defined as VPL of a feature that is relevant to a given task during training. TI-VPL is defined as VPL of a feature that is irrelevant to a given task [43]. In TI-VPL, learning has been found to occur in the absence of attention suggesting an early-level mechanism that supports learning. It was found that mere exposure to a stimulus feature that is task-irrelevant and sub-threshold was sufficient to induce learning [44]. Although TI-VPL has been found to occur without focused attention, it is subject to attentional inhibition if the irrelevant signals compete with the relevant signals [45]. Failure to suppress weak task-irrelevant or sub-threshold signals allow the non-suppressed signals to be learned. Additionally, learning could occur for task-irrelevant or task-relevant supra-threshold stimuli [46]. Although transfer of learning for untrained features was found, most TI-VPL studies have found learning to be specific to the feature that is trained. It is likely, then, that learning occurs at multiple levels of the system and may be sub-served by different mechanisms.

Despite the ability of the visual system to adapt to its changing environment, constraints are needed to protect the system from continued modification and change. Acquisition or gating of learning has been found to occur in one of two ways—through reinforcement or via attention. With reinforcement, learning occurs through spatially diffuse signals that enhance incoming sensory signals irrespective of whether the feature is task-relevant or task-irrelevant [43]. This form of passive learning, in which mere exposure to a stimulus within the visual field, facilitates both TR-VPL or TI-VPL. By comparison, attention selects what is behaviorally relevant and thus what is learned on a task. Attention can flexibly modulate cells involved in learning. For instance, monkeys performed a task at fixation while surrounded by previously trained stimuli [3]. The cells that responded when the previously trained stimuli were task-relevant were now being suppressed. This was thought to reflect that the previously trained stimuli were actively competing with the task-relevant stimuli now being presented at fixation. With regard to the necessity of attention in visual processing, it is important to note that the vast amount of information in the environment can overwhelm the brain’s limited processing capacity. One view of attention is that it is a selective mechanism by which aspects of information is prioritized over others thereby guiding learning and behavior. Two types of attention may be relevant for VPL—exogenous and endogenous attention.

Exogenous, or bottom-up, attention is a passive, transient, automatic, stimulus-driven process. Peripheral cues, presented near or at target stimuli, used to guide exogenous attention could be automatically captured by salient stimuli. With exogenous attention, feed-forward signals propagate from lower sensory areas to higher cognitive processing areas. Exogenous attention is driven by properties of a stimulus- such as color, orientation, luminance, can inadvertently go against the intentions of an observer [47–49], is deployed when salient novel stimuli are presented [50, 51] and is often difficult to ignore [52]. It can be triggered reflexively by a salient sensory event, such as a flash in the periphery. It works via signal enhancement of relevant signals [53, 54]. The time course of the shift of attention to an exogenous cue is approximately 100-120ms [55–57].

Endogenous, or top-down, attention is a voluntary, sustained, goal-driven process. Information that aligns with an observer’s behavioral goals are internally selected for further processing. It involves a more effortful process such as being instructed to orient attention to a particular location. The capture of attention is contingent upon top-down attentional control, a phenomenon known as contingent capture [58]. Posner [59] demonstrated an endogenous cueing paradigm in which participants *willfully* directed attention to a particular spatial location. A centrally presented symbolic cue, an arrow, would indicate the possible location of a subsequently presented target. The endogenous cue could point to the location of the target (valid trial), point away from the location (invalid trial), or give no indication to the location of the target (neutral trial). Performance is typically faster, more accurate, or both, for valid trials than for invalid trials and neutral trials. The source of endogenous attention is proposed to be through recurrent feedback connections that descend from higher cortical processing areas to lower sensory processing areas. Endogenous attention operates via signal enhancement of relevant signals and external noise reduction of irrelevant signals [53]. The time course of the shift of attention to an endogenous cue is approximately 300ms [60].

Although both types of attention share common perceptual effects [61, 62], each is capable of affecting information processing in distinct ways (for extensive list of unique perceptual effects see [60]). These two types of attention may differentially modulate perceptual processes. As a result, the effect on performance is likely to vary in accordance with the type of attention that is engaged. For instance, the benefits and costs in discriminability and processing speed differ between exogenous and endogenous attention [63]. For endogenous attention, benefits and costs increased with cue-validity whereas for exogenous attention, benefits and costs were constant across cue-validity. Unlike exogenous attention, endogenous attention can optimize performance according to task demands. For instance, endogenous attention was found to improve performance at all eccentricities by flexibly modulating resolution at attended locations [64]. In contrast, exogenous attention, regardless of the effect on performance, was found to automatically increase resolution at attended locations [65]. As a result, exogenous attention was found to improve performance at locations with low resolution but impair performance at locations with high resolution. In conditions of external noise, exogenous attention enhances contrast sensitivity under both low- and high- noise conditions whereas endogenous attention works under high-noise conditions [53, 54]. Whereas endogenous attention is susceptible to interference and requires more cognitive resources, exogenous attention does not [52, 66, 67]. The earliest stage of visual cortical processing is enhanced by exogenous attention whereas endogenous attention impacts later stages of processing [68]. Furthermore, endogenous attention may be maintained at a location for extended periods. On the other hand, exogenous attention exhibits inhibition of return, such that there is delay in responding from exogenous orienting after initial facilitation at same cued location [69].

Attention has often been argued as an important component of VPL. However, few studies have experimentally manipulated attention to examine its effects on VPL (see [70, 71, 21]). One study assessed varying effects of exogenous and endogenous attention on TR-VPL [72]. Both attentional cues were found to increase accuracy, but only exogenous attention was found to result in lower thresholds. Although this finding might suggest that the type of attention modulates VPL via distinct mechanisms, attentional cues were not systematically varied as participants were exposed to all validity cues, attended, divided-attended, and neutral, and thus make it difficult to assess the effect of attention. One such study did experimentally isolate the effect of exogenous attention on PL [33]. Learning was found when participants were trained with exogenous attentional cues as compared to neutral cues. Additionally, transfer of training was assessed for an untrained task and untrained feature and the findings indicated that learning only transferred to an untrained task. For location transfer, training with exogenous attentional cues facilitated transfer to untrained locations within and across visual hemifields whereas training with neutral cues exhibited location specificity [73]. A subsequent study, but with endogenous attention, also found transfer to untrained locations within and across visual hemifields whereas training with neutral cues exhibited location specificity [74]. Specifically, the study investigated whether, like exogenous attention, endogenous attention facilitates learning and location transfer in an orientation discrimination task. Notably, performance improvements as a result of training with attention carried over to untrained locations despite the effect of attention being local to the target location.

Despite similar performance improvements across both studies, distinct mechanisms underlie facilitation of location transfer with exogenous attention operating via response gain [73] and with endogenous attention operating via contrast gain [74]. Performance changes in response to changes in stimulus intensity is characterized by the psychometric function. Changes in the psychometric function via contrast or response gain are thought to reflect neuronal activity as a function of stimulus contrast, which creates a contrast response function [75]. Attentional modulation via contrast gain leads to an increase in contrast sensitivity with no change in the relative firing rate. Contrast gain may be reflected in the psychometric function as a left-ward shift in the psychometric function. Whereas, response gain leads to a multiplicative increase in firing rate across the contrast response function with no change in threshold. Response gain may be reflected behaviorally as improved accuracy across stimulus intensity with pronounced effects as higher stimulus intensities. Another study investigated the role of exogenous attention in the transfer of learning across location in two different acuity tasks; Landolt acuity and Vernier acuity [76]. In the Landolt acuity task, training with exogenous cues resulted in location transfer whereas, training with neutral cues resulted in location specificity. However, in the Vernier acuity task, training with both exogenous and neutral cues resulted in location specificity. This suggests that even after training with exogenous attention, learning on certain tasks may not be amenable to transfer across locations. Altogether, these findings demonstrate the effect of the type of attention, albeit complicated, on learning and specificity.

The extensive literature on PL has resulted in several different models that consider the role of attention. These models differ in terms of several different factors that include what cortical area is activated, the degree of specificity of VPL, and the type of VPL. For the purpose of the present study, VPL models that explicitly incorporate attention will be discussed. The Reverse Hierarchy Theory [14, 77] assumes learning is a top-down attention-guided process. According to this model, the locus of learning are higher processing areas that allow for learning on tasks that involve coarse discrimination. Learning, then, cascades to lower processing areas for tasks that require fine discrimination. Specificity arises with continued training allowing access to lower level processing sites. This model assumes that top-down attention is required for the occurrence of VPL and thus does not account for findings regarding TI-VPL.

A more recent theory, the Dual-Plasticity model, posits learning can occur in the presence of or absence of attention [30, 78]. This theory proposes two types of plasticity; task-based plasticity and feature-based plasticity. Feature-based plasticity is defined as changes in the representation of features. Task-based plasticity is defined as changes in processing related to a trained task. Feature-based plasticity results from mere exposure to a feature during training irrespective of whether the feature is task-relevant or task-irrelevant and is specific to the exposed feature. On the other hand, task-based plasticity stems from involvement on a trained task, occurs only in TR-VPL, and is specific to the trained task. According to this model, changes associated with feature-based plasticity should be observed as changes in neural responses to the trained feature (in the corresponding visual areas in association with TR-VPL) as opposed to task-based plasticity which will be associated with changes in high-level cognitive areas or connectivity between visual and cognitive areas. Evidence for two types of plasticity, that occur in different cortical regions, has been found suggesting that, to some degree, separate mechanisms exist [79].

The purpose of the current study was to examine the role of different types of attention in TR-VPL. Specifically, the present study investigated the role of exogenous and endogenous attention on TR-VPL. Within the framework of the dual-plasticity model, it was hypothesized that endogenous attention is important for TR-VPL and exogenous attention is not important for TR-VPL. This suggests that in the present study, learning will be enhanced when endogenous attention, as compared to exogenous attention, is engaged. Furthermore, learning will be impacted by cue-validity with greater learning for higher cue-validity conditions as opposed to lower cue-validity conditions when endogenous attention is engaged. Finally, the issue of transfer of learning to an untrained stimulus was examined. If transfer of training occurs, then transfer should be greater for endogenous attention conditions as compared to exogenous attention conditions because of the greater role of endogenous attention in TR-VPL.

## Methods

### Participants

60 college-aged adults (mean age = 19 years, SD = 2.35) from the University of California, Riverside (35 male and 35 female) participated in the study. All participants were compensated for their participation at a rate of $15 per hour. The Institutional Review Board of University of California, Riverside approved this study. Participants gave their written informed consent for their participation and participants were debriefed on the purpose of the study after their participation. All participants had normal or corrected-to-normal visual acuity and were naive to the purpose of the study. Participants’ near visual acuity was screened using a LogMAR chart (*M* = -0.05, *SD* = 0.08). All participants were screened for eye diseases through self-report. Corrective lenses normally worn by the participants were allowed during the experiment. Participants with extreme initial contrast thresholds prior to training -/+1.5 SD were excluded from the study. Seven participants were excluded from the study due to consistently high thresholds indicating that they could not perform the task.

### Apparatus

Stimuli were presented on a 49.53-cm CRT monitor Viewsonic PF817 at a resolution of 1,024 × 768 pixels; the monitor had a refresh rate of 75 Hz non-interlaced and a mean luminance value of 42.7 cd/m^2^. Stimuli were generated on an Alienware AREA_51 PC equipped with an Intel Core i7 960 processor using the Windows 7 Ultimate 2009 operating system. A NVIDIA GeForce GTX 480 graphics card was used along with a Bits++ system (Cambridge Research Systems, Rochester, Kent, United Kingdom) to achieve 14-bit gray scale (16,384 gray-scale levels). Custom experimental software was written in MATLAB (The MathWorks, Natick, MA); Psychophysics Toolbox extensions (Brainard, 1997; Pelli 1997; Kleiner, Brainard & Pelli, 2007). The EyeLink 1000 Tower Mount (SR Research, Ottawa, Ontario, Canada) was used to monitor participants’ eye movements as well as stabilize head position. The monitor was calibrated using a ColorCal 2 colorimeter (Cambridge Research Systems).

Participants’ far acuity was measured using the 2000 Series Revised ETDRS Chart 2 (Precision Vision, La Salle, IL) at a distance of 3 m. Participants’ near acuity was measured using the 2000 series New ETDRS Chart 3 at a distance of 40 cm. Contrast sensitivity was measured using the Pelli-Robson Contrast Sensitivity Chart (Precision Vision).

### Stimuli and procedure

The stimulus was a gabor patch defined by a sine wave at a spatial frequency of 0.5 cycles/degree of visual angle and the target was the gabor patch but with the presence of an additional sine wave at a spatial frequency of 1.5 cycles/degree [22, 80]. Contrast of the 0.5 cycle/degree sinusoid is denoted as c_1_ and the contrast of the 1.5 cycles/degree sinusoid, c_2_. Gabor stimuli were embedded in fixed additive Gaussian noise with a standard deviation set at 0.33 throughout the experiment. The phase of the stimuli was randomized ±180° on each trial. Luminance was matched across trials using root-mean-square luminance [81]. Contrast thresholds were derived.

Stimuli were presented at one of two locations; left or right 7.5 degree of visual angle from center. Presentation of the gabor patch at one location was always accompanied by its corresponding distractor stimulus at the opposite location. The distractor stimulus was the same gabor patch presented during the trial but each pixel at each location that composed the gabor patch was randomized to a new location. This allowed for constant luminance on each trial. All stimuli were enveloped by a Gaussian mask with a standard deviation of 2.2 degrees of visual angle.

To remove any edge cues, all stimuli were viewed through a black circular aperture with a radius of 2.4 degrees of visual angle and aperture thickness of .4 degrees. In order to assess transfer, stimuli were presented at two orientations; 20 degrees and 110 degrees.

The task was a Yes/No detection task in which participants were to determine the presence or absence of an additional sinewave component in the gabor patch. Participants were to respond on the numeric keypad with ‘1’ if the additional 1.5 cycle/degree sine-wave was present or ‘2’ if additional 1.5 cycle/degree sine-wave was not present. A fixation point was a bulls-eye target with a radius of .8 degrees of visual angle presented in the center of the screen. Participants were to maintain fixation on the center bulls-eye target throughout the experiment. The eye tracker was utilized to ensure participants were fixated on the center. Given the temporal nature of endogenous cues and that ~200-250ms are needed for goal-directed saccades, stimulus onset asynchrony (SOA) for the endogenous cue may allow participants to make an eye movement [82]. To control for this, trials would restart if participants were not fixated on the center.

**Fig 1:**
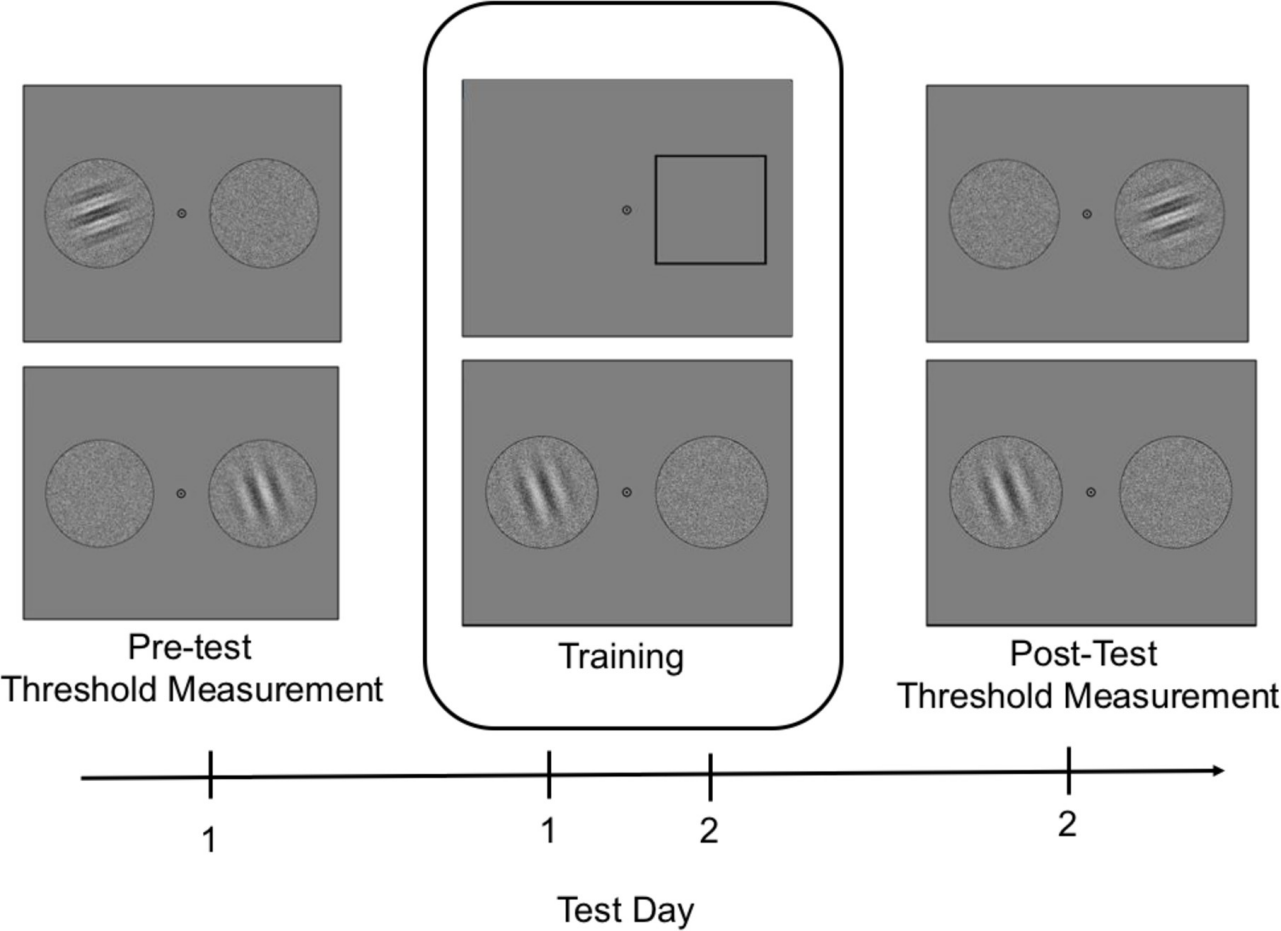
Time course of the experiment. Contrast thresholds for trained and untrained orientation were obtained at 75% correct. Training sessions occurred on day 1 and day 2 after pre-test and prior to post-test. Attention (exogenous, endogenous) and cue-validity (100%, 80%, neutral) were manipulated at training.

The experiment was administered over 2 consecutive days with 1 hour each day. Each day consisted of 2 sessions: on day 1, contrast threshold measurements were obtained prior to training then one training session with attentional cues while on day 2, one training session with attentional cues then contrast threshold measurements were obtained after training. Stimuli were viewed binocularly on a monitor at a distance of 95.25 cm. The experiment was run in a darkened room; the only light source was the monitor. Refer to Fig 1 for the time course of the experiment.

### Practice

Before the start of the experiment and at the start of day 1, all participants were given 4 blocks of 20 trials to familiarize them to the task. Orientation of the stimuli was alternated across each block. At the start of each trial, participants saw a fixation point in the center of the screen for 1000-ms followed by a 1000-ms presentation of the stimuli (simultaneous presentation of gabor stimuli and noise stimuli). Following the presentation of the stimuli, a uniform mid-gray background was presented to indicate that subjects should respond. After the response cue, feedback was given based on their response. Stimuli were presented for 1000-ms for the first two blocks and then for 53-ms for the last two blocks. For practice trials, contrast values were fixed at c_1_ = 0.6 and c_2_ = 0.4; values that were well above threshold.

### Testing

Contrast thresholds of c_2_ were assessed for trained and untrained orientations at the beginning of day 1 and the end of the day 2 of the experiment. c_1_ was set at a fixed value of 0.4. The QUEST procedure was used to measure contrast thresholds of contrast c_2_ of the gabor patch [83]. QUEST was initialized with a criterion level of 0.75 (β = 1.3, δ = 0.10, γ = 0.5). This β value is based on a preliminary study designed to find the optimal β value for the task. Participants completed 60 trials/block for 4 blocks. The first two blocks were presented at one orientation and the last two blocks were presented at another orientation. 60 trials were collected at each orientation for contrast c_2_ of the 1.5 cycles/degree sine-wave in the gabor patch. From preliminary data, 60 trials for each stimulus orientation was sufficient for QUEST to converge at a stable threshold estimate.

A fixation point was presented for 53-ms followed by a 53-ms presentation of the stimuli (simultaneous presentation of gabor patch and noise stimuli). Then, the response cue followed by feedback based on their response. Testing order of the orientations were counterbalanced across participants. Participants were given a one-minute break after each block. During testing, stimuli were presented without cues.

### Training

Training occurred on day 1 after testing and on day 2 prior to testing with a 5-minute break between testing session and training session. Using the same stimuli and a similar procedure as in the testing phase- with the key difference being that participants were trained on only one orientation instead of the two they saw during testing sessions. During training, all subjects completed six blocks with 60 trials each block. Subjects were given a one-minute break after each block.

During each training session, the QUEST procedure was run using the same parameters as during the testing sessions, but with one modification. The contrast threshold estimate for QUEST for each subject used the threshold derived on the prior session as the initial estimate. Subsequent trials used the current threshold, taking into account each trial during the training sessions. Using this method, subjects were trained at their threshold (75% correct) across all training trials. Of the 720 trials administered over the course of the training sessions, only 360 trials were inputted into QUEST to derive contrast thresholds.

To assess the effectiveness of attention on PL, participants were divided and trained in one of three cue-validity conditions; 100% valid in which the cue always specified the location of the target, 80% valid in which the cue specified the location of the target 80% of the time with the other 20% invalid in which the cue specified the location of the distractor, and a neutral condition in which the cue specified both locations. For the training sessions, the attentional cues preceded the simultaneous presentation of the gabor and noise stimuli. Following Posner’s [59] original experiment, the neutral cue condition of the endogenous group displayed a fixation cross rather than a double-ended arrow.

To assess possible differential effects of the type of attention on PL, one group was trained with an exogenous attentional cue (n = 30) and another group was trained with an endogenous attentional cue (n = 30). From the type of attention, these participants were further divided into the three cue-validity conditions with 10 participants in each condition. The exogenous attentional cue was a visually salient cue (white square-shaped frame) presented at either one of the two locations (100% or 80% valid) or both locations (neutral) [48]. The endogenous cue was a black arrow presented in the center of the screen that could point to one of two locations (100% or 80% valid) or both locations (neutral) (adapted from [59]). In regard to both types of cues, participants were instructed that the presence of the cues, either a ‘white-square outline’ for the exogenous cue or ‘black-arrow’ for the endogenous cue, prior to the presentation of the stimuli may or may not indicate the location of the target pattern.

**Fig 2:**
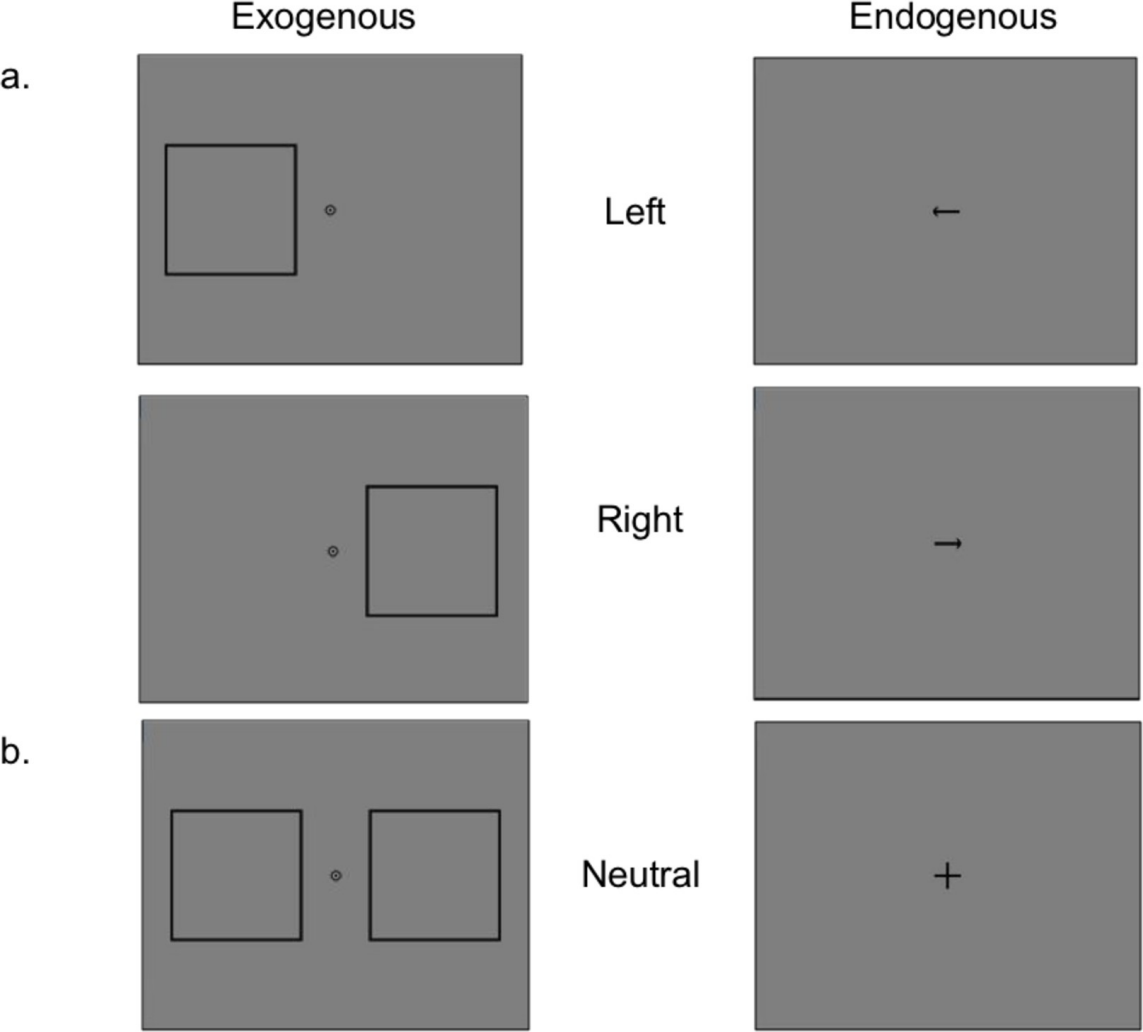
Attention cues (exogenous, endogenous) used during training. Left column shows exogenous cues, which were presented for 67ms. Right column shows endogenous cues, which were presented for 305ms. (a) left indicating (top-row) and right indicating (bottom-row) cues for either valid/invalid trials presented for 100% and 80% cue-validity condition. (b) neutral cues were presented only for the neutral cue-validity condition.

The time course for a training trial was as follows: a fixation point was presented for 1000-ms followed by either one of two attentional cues (67-ms for an exogenous cue and 305-ms for an endogenous cue). The endogenous cue was a black arrow (2 degrees for length of the arrow and .5 degrees for the arrow hands) presented near the center of the fixation point. The exogenous cue was a white square outline (10 degrees in length for each side of the square) presented near the stimuli. Next, an inter-stimulus interval (ISI) was presented for 53-ms followed by the training stimuli (simultaneous presentation of the gabor and noise stimuli) which was presented for 53-ms. After the stimuli disappeared, a response cue indicated to participants to make a judgment followed by feedback. Refer to Fig 2 for attentional cues used during training.

## Results

**Fig 3:**
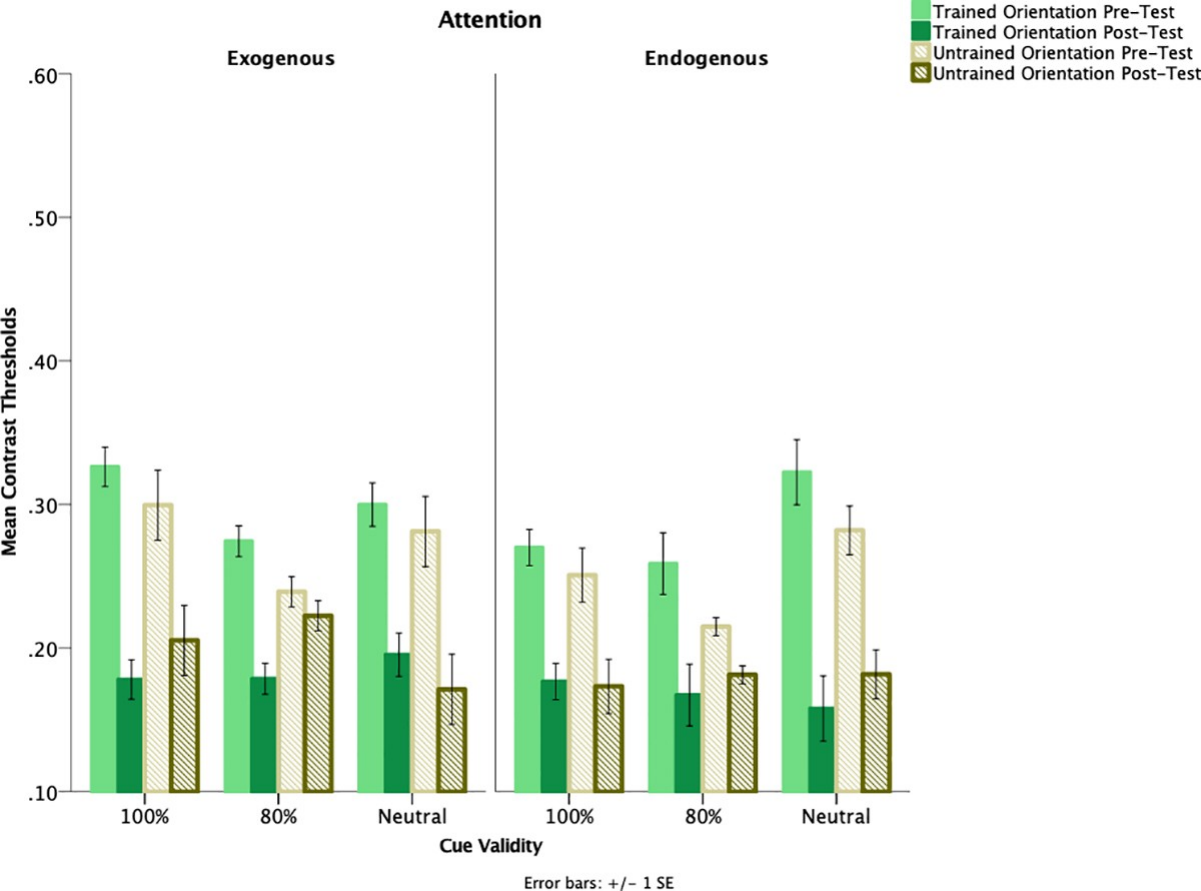
Changes contrast thresholds measured at pre-test and post-test. Contrast thresholds measured at pre-test and post-test for both trained and untrained orientation as a function of attention and cue-validity. Error bars indicate +/- 1 within-subjects standard error.

Contrast thresholds prior to training were examined using a 2 (attention: exogenous, endogenous) x 3 (cue-validity: 100%, 80%, neutral) x 2 (feature orientation: trained, untrained) mixed–design analysis of variance (ANOVA). Attention type and cue-validity were between-subject factors. Orientation was a within-subjects factor. Analyses were conducted using IBM SPSS Statistics software Version 24. The first analysis conducted was to assess whether there were any differences between the exogenous and endogenous attention groups prior to training. This was assessed by examining the type of attention and thresholds for the trained and untrained orientation prior to training. Differences prior to training were found across attention and cue-validity conditions for both trained orientation, and untrained orientation, *F*(1,54) = 4.716, *p* = .034 η_p_^2^ = .080 with higher thresholds for trained orientation (*M* = .292, *SE* = .016) as compared to untrained orientation (*M* = .261, *SE* = .016). Refer to Fig 3 for contrast thresholds at pre-test and post-test.

### Magnitude of learning

As indicated above, given differences in initial performance, and in order to compare performance across attention type, a magnitude of learning score was calculated for trained and untrained orientation by calculating the difference between pre-test threshold from post-test threshold and then divided by the pre-test threshold.

$$Magnitude\;of\;learning=\frac{PostTest\;Threshold-PreTest\;Threshold}{PreTest\;Threshold}$$

By doing so, this allows for an examination of any changes in performance given an individual participant’s initial performance level. Using this metric, it was found that there was a significant difference between trained and untrained orientation, *F*(1,54) = 15.627, *p* < .001, η_p_^2^ = .224 with greater magnitude of learning for trained orientation (*M* = -.352, *SE* = .023) as compared to the untrained orientation (*M* = -.201, *SE* = .039). There was a non-significant trend for the effect of cue-validity, *F*(2,54) = 2.590, *p* = .084, η_p_^2^ = .088.

**Fig 4:**
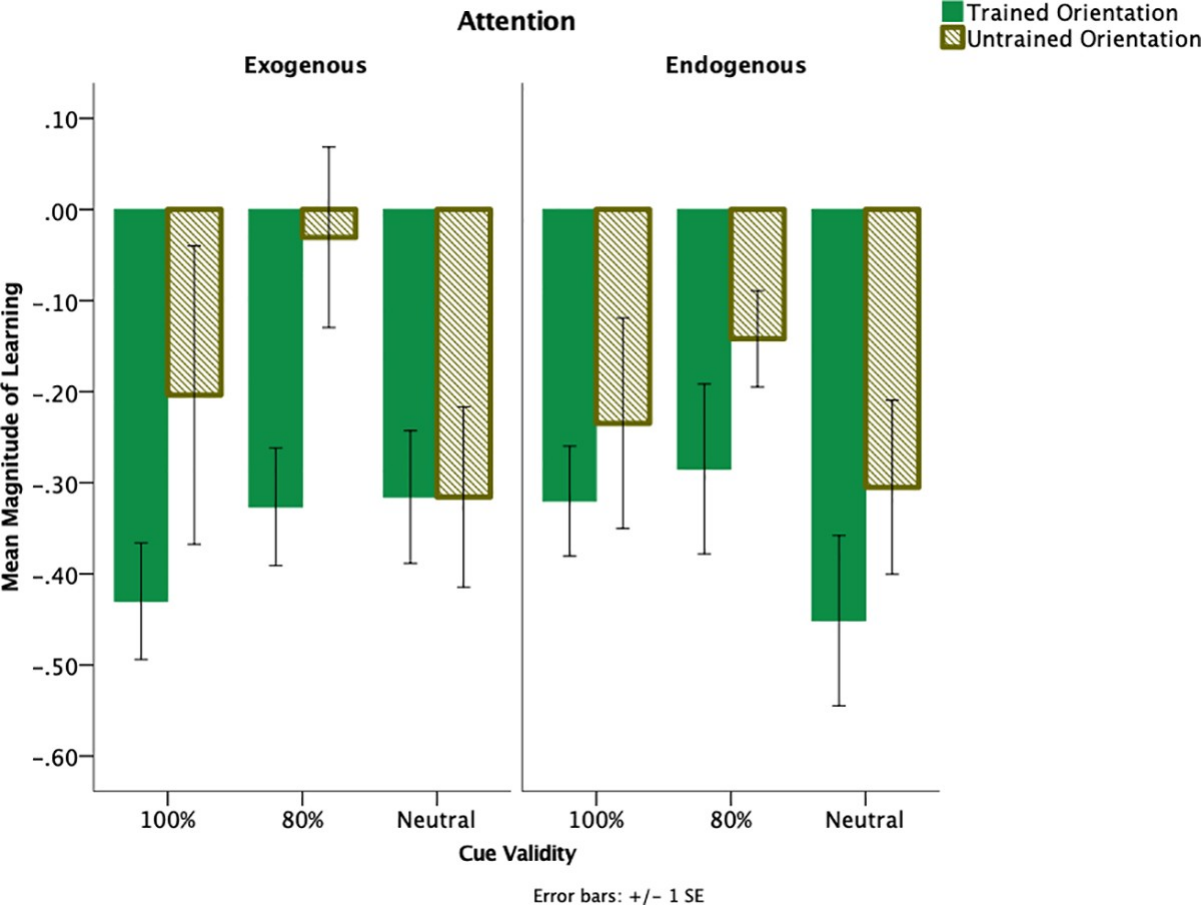
Magnitude of learning as a function of cue-validity and attention. Larger negative values indicate greater learning. Error bars indicate +/- 1 within-subjects standard error.

To assess whether magnitude of learning differs by attention type and cue-validity, a 2(attention: endogenous, exogenous) x 3 (cue-validity: 100% valid, 80% valid, neutral) factorial ANOVA was conducted. No interaction between attention and cue-validity on magnitude of learning for trained orientation was found, *F*(2,54) = 2.080, *p* = .135, η_p_^2^ = .072. The results for magnitude of learning across attention groups is shown in Fig 4. Subsequent analyses were conducted separately by attention type.

### Exogenous attention

A cue-validity (100%, 80%, neutral) x feature orientation (trained, untrained) x test day (pre-test, post-test) mixed-design ANOVA was conducted on correct response times (RT). Test day was a within-subjects factor. There was an effect of test day such that participants were faster following training from pre-test (*M* = .728, *SE* = .041) to post-test (*M* = .602, *SE* = .017), *F*(1,27) = 16.555, *p* < .001, η_p_^2^ = .380. Thresholds prior to training reveal performance was comparable across cue-validity conditions, *F*(1,27) = 1.951, *p* = .174, η_p_^2^ = .067. A 2 (feature orientation: trained, untrained) x 3 (cue-validity: 100%, 80%, neutral) x 2 (test day: pre-test, post-test) mixed–design ANOVA was conducted. There was a main effect of test day, *F*(1,27) = 32.210, *p* < .001, η_p_^2^ = .544 such that post-test thresholds (*M* = .192, *SE* = .011) were significantly lower than pre-test thresholds (*M* = .287, *SE* = .020). There was no main effect of orientation, *F*(1,27) = .265, *p* = .611, η_p_^2^ = .010 suggesting that there were no differences between trained and untrained orientation.

There was also a non-significant trend of an interaction between orientation and test day, *F*(1,27) = 3.514, *p* = .072 η_p_^2^ = .115, such that training improved thresholds (trained orientation: post-test threshold, *M* = .184, *SE* = .011; pre-test threshold, *M* = .300, *SE* = .021) as opposed to the untrained thresholds (untrained orientation: post-threshold, *M* = .200, *SE* = .014;pre-test threshold, *M* = .273, *SE* = .023). Consistent with our hypothesis, there was no effect of cue-validity, *F*(1,27) = .265, *p* = .776, η_p_^2^ = .019.

### Endogenous attention

A cue-validity (100%, 80%, neutral) x feature orientation (trained, untrained) x test day (pre-test, post-test) mixed-design ANOVA was conducted on correct response times (RT). There was an effect of test day such that participants were faster following training from pre-test (*M* = .762, *SE* = .083) to post-test (*M* = .575, *SE* = .015), *F*(1,27) = 5.800, *p* = .023, η_p_^2^ = .177. Thresholds prior to training reveal performance was comparable across cue-validity conditions, *F*(1,27) = 2.778, *p* = .107, η_p_^2^ = .093. A 2 (feature orientation threshold: trained, untrained) x 3 (cue-validity: 100%, 80%, neutral) x 2 (test day: pre-test, post-test) mixed–design ANOVA was conducted. There was a main effect of test day, *F*(1,27) = 29.401, *p* < .001, η_p_^2^ = .521 such that post-test thresholds (*M* = .173, *SE* = .009) were lower than pre-test thresholds (*M* = .267, *SE* = .020). There was an interaction of trained orientation x test day, *F*(1,27) = 4.931, *p* = .035, η_p_^2^ = .154 such that thresholds improved from pre-test (*M* = .283, *SE* = .023) to post-test(*M* = .167, *SE* = .011) of the trained orientation as compared to the pre-test(*M* = .249, *SE* = .021) and post-test of the untrained orientation(*M* = .179, *SE* = .009). There was no effect of orientation, *F*(1,27) = .860, *p* = .362, η_p_^2^ = .031. Contrary to our hypothesis, there was no effect of cue-validity, *F*(2,27) = .495, *p* = .615, η_p_^2^ = .035.

### Training sessions

**Fig 5:**
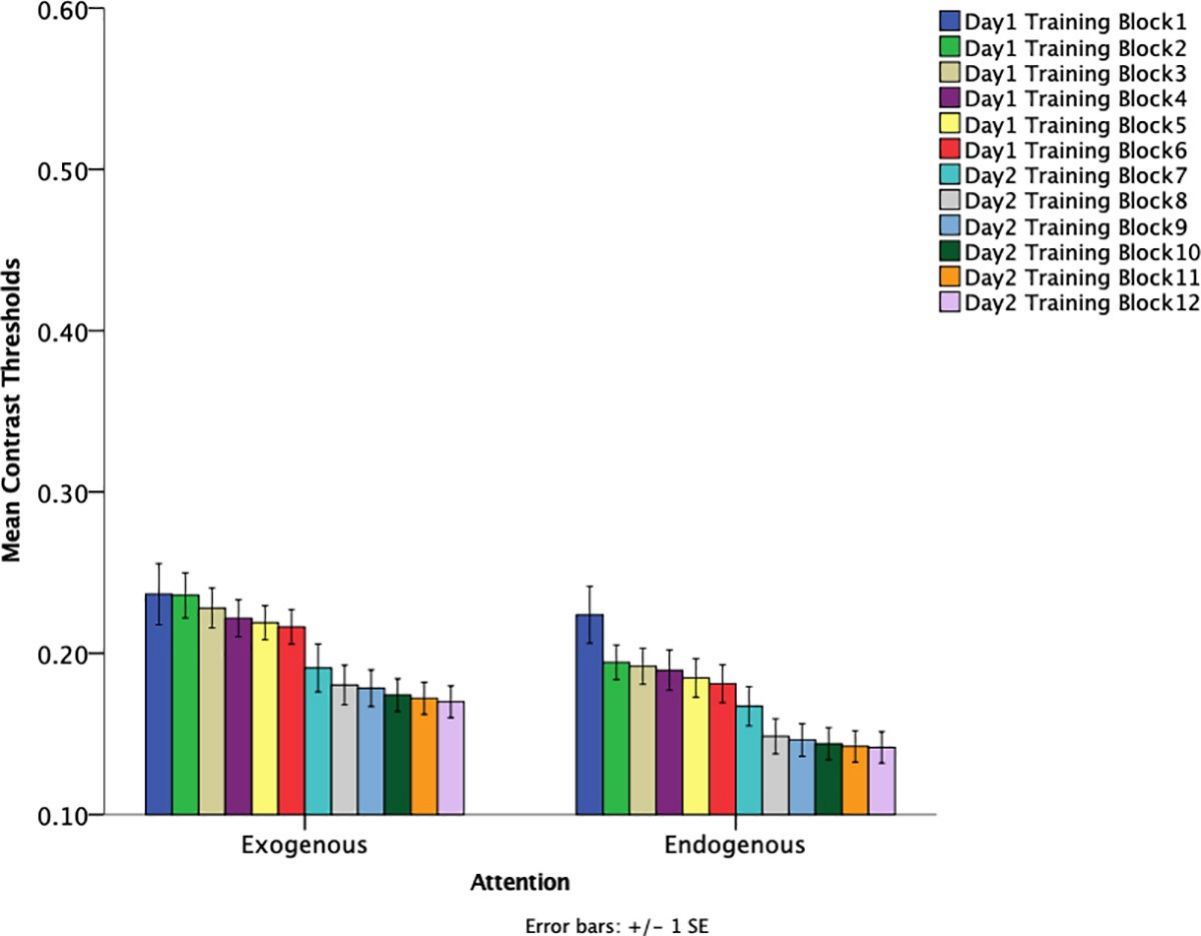
Mean contrast thresholds for training data as a function of attention. Blocks 1–6 were on training day 1 and blocks 7–12 were on training day 2. Error bars indicate +/- 1 standard error.

**Fig 6:**
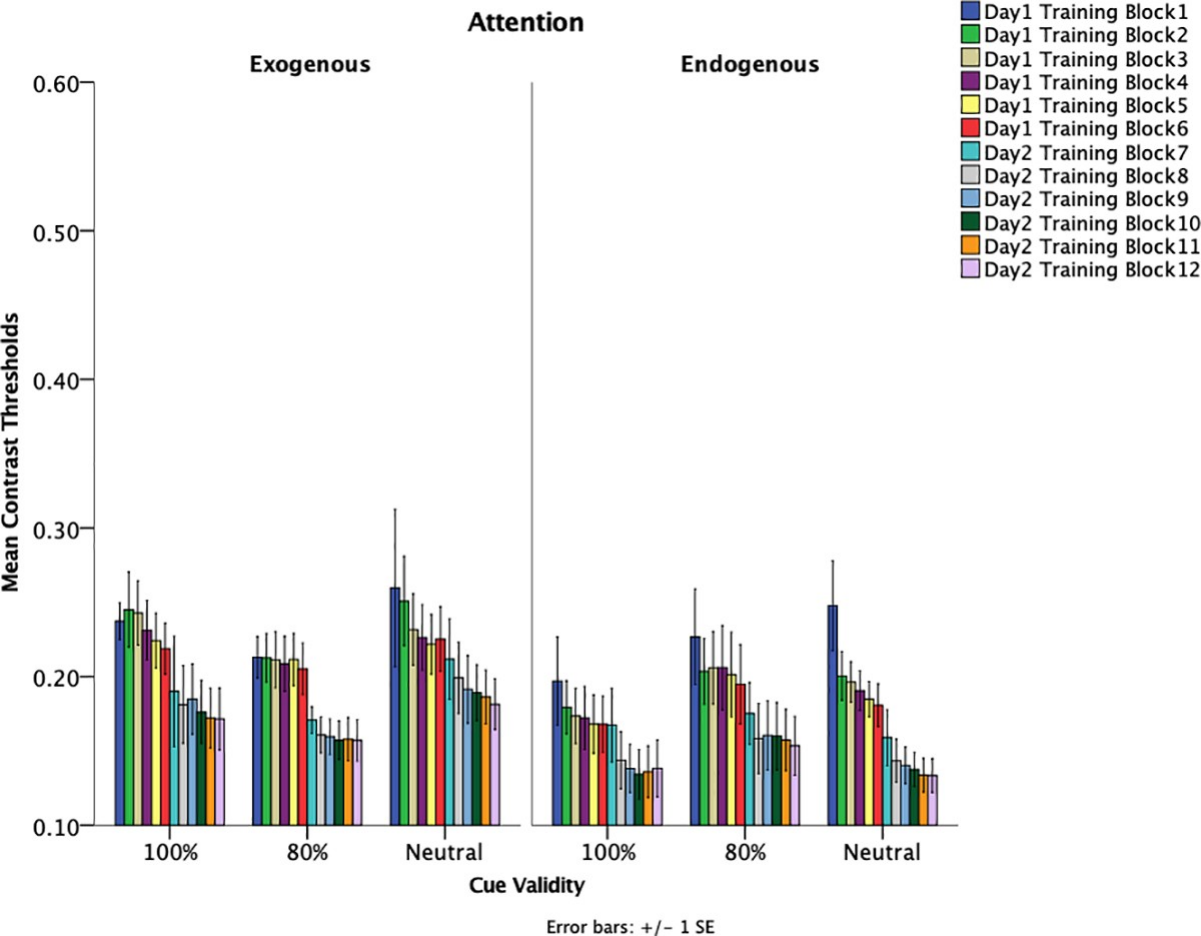
Mean contrast thresholds for training data as a function of cue-validity and attention. Blocks 1–6 were on training day 1 and blocks 7–12 were on training day 2. Error bars indicate +/- 1 standard error.

If any attentional differences exist, then such differences may be present as a function of training sessions. To investigate whether any differences occurred during training, contrast thresholds were obtained at the end of each training day. Contrast thresholds were examined using a 2 (attention type: exogenous, endogenous) x 3 (cue-validity: 100%, 80%, neutral) x 2 (training day: day 1, day 2) mixed–design ANOVA. Training day was a within-subjects factor. An effect of attention was found such that contrast thresholds of those in the endogenous attention conditions had significantly lower thresholds (*M* = .161, *SE* = .010) for both training days as compared to the exogenous attention condition (*M* = .193, *SE* = .010), *F*(1,54) = 4.880, *p* = .031, *η*_*p*_*2 =* .083. Fig 5 shows training data as a function of different attention groups. These results suggest overall enhanced learning when endogenous attention, as compared to exogenous attention, is engaged during training. This may stem from learning-induced changes in both sensory feature representations and task-related processing. There was an effect of training day such that the second day of training (*M* = .156, *SE* = .007) had significantly lower thresholds than the first day of training (*M* = .199, *SE* = .008), *F*(1,54) = 131.11, *p* < .001, *η*_*p*_*2 =* .708. Not only does it appear that training facilitated learning but sleep after a session of training appears to enhance learning, which suggests memory consolidation. Specifically, there is a noticeable decrease in thresholds from training day 1 to training day 2. As shown in Fig 6, this effect can be seen when examining the first block of the last training session (day 2, training block 7) and the last block of the first training session (day 1, training block 6). An analysis on the last block of the first training session (*M =* .199, *SE* = .008) and first block of the last training (*M* = .179, *SE* = .010) session reveal a significant decrease in threshold, *F(*1, 54) = 8.232, *p* = .006, *η*_*p*_*2* = .132.

### d’ (sensitivity)

For each participant, a d’ (measure of sensitivity/detection) was calculated. One participant did not produce a valid d’ measure due to high correct rejection rate and so a correction was applied to the participant’s data to obtain a valid d’ measure [84]. To investigate whether there were any baseline group-level differences in sensitivity (d’), prior to training, between trained and untrained orientation, a 2(attention type: exogenous, endogenous) x 3(cue-validity: 100%, 80%, neutral) x 2(feature orientation: trained, untrained) mixed design ANOVA was conducted on d’ measures. There was no effect of feature orientation, *F*(1,54) = 2.619, *p* = .111, η_p_^2^ = .046. Additionally, no 3-way interaction was observed, *F* (2,54) = .201, *p* = .818, η_p_^2^ = .007. This suggests no differences in sensitivity prior to training between trained and untrained orientations across all conditions.

Subsequent analyses allowed for comparisons across all groups (attention type (exogenous, endogenous) x cue validity (100% valid, 80% valid, neutral)). Changes in sensitivity across testing day was conducted using 2(attention type: exogenous, endogenous) x 3(cue-validity: 100%, 80%, neutral) x 2(feature orientation: trained, untrained) x 2(test-day: pre-test, post-test) mixed design ANOVA. There was an effect of orientation with greater sensitivity for trained orientation (*M* = 1.721, *SE* = .056) as compared to untrained orientation (*M* = 1.595, *SE* = .058), *F* (1,54) = 4.152, *p* = .046, η_p_^2^ = .071.

## Discussion

The current study was concerned with investigating differential effects of exogenous and endogenous attention on TR-VPL on a novel yes/no detection task that involves determining the presence or absence of an additional sine-wave component in the gabor stimulus. We used this type of discrimination task because previous research has found rapid improvement with practice for this type of stimuli [23]. Analyses on pre-test contrast thresholds for trained and untrained orientation across cue-validity condition and attention type show that participants were not at similar levels of performance prior to training. High inter-observer variability is commonly found in the field of PL [16, 85–88]. In fact, not only are individual differences in initial performance levels common in the field of PL but also the rate of learning is variable among participants.

An analyses of attention x cue-validity x test day was problematic because of different baseline performances between the groups prior to training. For example, a group that performed well, prior to training, may already be at asymptotic performance with minimal range for improvement. In contrast, a group with very poor thresholds is likely to have a greater range for improvement. As a result, separate analyses were conducted based on the attention condition. For exogenous attention, there was an overall improvement of thresholds following training. When analyses were conducted separately by cue-validity condition, learning was observed across all cue-validity conditions for the trained orientation but these improvements did not transfer to the untrained orientation. This suggests that learning was specific to the trained feature when trained with exogenous cues, regardless of cue-validity. For the endogenous condition, different patterns of performance were observed between the cue-validity conditions. For the 100% cue-validity, learning was specific to the trained orientation. For the 80% cue-validity condition, learning was not observed for the trained orientation but performance improvements were observed for the untrained orientation. For the neutral condition, both learning and transfer was observed.

An alternative approach was to evaluate the magnitude of learning. Thus, any changes in performance could be evaluated with respect to an individual participant’s initial performance level. There was a significant difference in the magnitude of learning between trained and untrained orientation. Improved thresholds following training were found across exogenous and endogenous attention regardless of cue-validity conditions. Moreover, the magnitude of improvement between exogenous and endogenous attention was comparable. This is consistent with previous studies that found both exogenous and endogenous attention facilitated both learning and transfer but evaluated in the context of learning across locations in an orientation discrimination task [73, 74]. The current study failed to find behavioral differences between exogenous and endogenous attention. It is possible that both forms of attention may lead to similar levels of improvement at the behavioral level. But the dissociation between endogenous and exogenous attention may stem from different mechanisms involved to achieve changes in performance. Previous studies demonstrated that training with either valid exogenous [73] or valid endogenous cues [74] both facilitated learning and location transfer. Despite similar performance improvements, distinct neural signatures underlie exogenous and endogenous attention with performance improvements for exogenous attention achieved via response gain [73] and for endogenous attention was achieved via contrast gain [74]. Threshold measurements employed in the current study may not estimate changes in performance to the same degree as accuracy measurements typically employed in PL studies. Previous research evaluating contrast thresholds and accuracy did not find any correlation between the behavioral changes observed during training and improvements in contrast sensitivity [72]. This study provides additional evidence that threshold measurements may be qualitatively different from accuracy measurements in the context of PL.

### Cue-validity

It was predicted that greater learning would occur for higher cue-validity conditions when endogenous attention was engaged. We did not find an interaction between attention and cue-validity on the magnitude of learning. Performance was comparable across attention type and cue-validity condition. This suggests that magnitude of learning does not vary with cue-validity when endogenous attention is engaged, which is at odds with previous findings [63]. The failure to find an effect could be due to the type of stimulus training. We utilized a task in which participants were presented with complex sine wave patterns. This task was used because it results in rapid learning. The current study trained participants over the course of 2 days for several hundred trials. This differs from a majority of PL studies that often train participants for several sessions and thousands of trials in order to produce training-related learning. However, few studies have employed fewer training sessions and were able to produce learning [22–24, or see 31, for a review]. Particularly relevant to the current study is that learning has been observed following a few hundred trials of training for complex grating patterns [22–23]. Studies that have examined the time course of learning have shown at least two different learning processes; rapid and slow learning [89]. Rapid learning occurs over a few hundred trials, affects higher-level processing, exhibits generalized learning, and may involve top-down processing by improving the link between task-dependent processing and sensory units while selecting optimal units for the task [2]. In contrast, slow learning is thought to be a slower process that occurs over several hundred trials or more, exhibits stimulus specific learning, and may involve lower-level processing by modulating primary sensory areas. It is possible that the failure to find a training difference based on the type of attention was due to the use of stimulus conditions that result in rapid learning as compared to slow learning. An important question will be to examine this question in future research.

### Feature transfer

Contrary to our hypothesis that feature transfer of training would occur via endogenous attention only, the results indicate feature transfer occurred for both exogenous and endogenous attention. This is not necessarily at odds with the Dual-Plasticity model [30, 78]. According to the Dual-Plasticity model, task-related processing involves both feature and task-based plasticity whereas feature-related processing involves feature-based plasticity. Given that both endogenous and exogenous attention may involve feature-based plasticity, transfer to an untrained feature may have occurred for both forms of attention. And so, any performance differences may not have been apparent because both attention types may have engaged similar mechanisms on the feature transfer task. Future research should focus on learning and transfer across different tasks between exogenous and endogenous attention and whether there is a differential effect of attention on transfer to an untrained task.

A caveat to this study is that it cannot rule out that feature transfer may be due to the novel paradigm employed. Generally, in the field of PL, performance improvements tend to be specific to the trained feature with performance improvements lost when stimulus feature or stimulus location has changed. Exceptions to this finding are studies that employ the double-training or training-plus-exposure technique [35, 90–92] or introducing variability in stimulus location or stimulus set, or exposure to an untrained location (see [31], for a review).

To account for conditions under which transfer occurs, the integrated reweighting theory posits that transfer to new retinal positions/locations is fundamentally different from transfer over stimulus features [40]. Location transfer is proposed to be mediated by location-independent representations whereas feature transfer reflects the compatibility of the weight structures between location-specific and location-independent representations. According to this framework, transfer is predicted when the same stimulus feature is presented in a new location and specificity is predicted when a new stimulus feature is presented to the same trained location. The study by Dosher and colleagues [40] examined the extent of transfer by training observers on an orientation discrimination task and subsequently randomly assigned participants to one of three conditions. Participants were either randomly assigned to continue training either in a condition in which the same stimulus feature was presented in a new location/position (P), a new stimulus feature was presented (O), or both a new orientation and new location was presented (OP). Greater transfer was found when the same stimulus feature was presented in a new location (P) than a new stimulus feature presented in the same location (O) [40]. But there was partial transfer when both a new stimulus feature and new location was presented. In the current study, the location of the target stimulus varied by trial and exposure to the untrained orientation in combination with rapid learning for the type of stimuli used may have enabled transfer of learning to the untrained orientation. Finding of generalization across untrained feature is more compatible with the view that learning occurs at a higher level of neuronal plasticity.

How might these findings fit within the broader context of attention and PL? The results of the present study suggest that the utility of attention in PL may depend on a variety of factors. For instance, the task configuration was optimal for targeting the early component processes involved in rapid learning. In studies involving rapid learning, the findings of specificity or transfer may well depend on the neural structures involved in the training task. With rapid learning, training of simple visual discriminations was found to be specific [31, 23] but training with visual search, which presumably involves neural structures further along the visual hierarchy, was found to transfer [93]. Additionally, the rapid improvements found in the current study may reflect an early component learning process that have been shown to exhibit generalized learning. Previous findings reported generalized learning in an early phase of training followed by specificity of learning [32, 42]. These studies are consistent with two qualitatively distinct component processes of learning; rapid and slow learning. This has important implications for training studies involving some marked visual dysfunction such as those with amblyopia. The source of dysfunction in amblyopes is loss of critical information in early visual processing. Thus, any effective intervention should restrict the site of training to target those neural structures, be broad enough to generalize learning, require little intervention yet maximal benefit, as well as minimal effort from the individual.

Whether specifically manipulating attention further enhances learning may require a closer look at the type of task utilized. It seems whether attention is distributed (as in the neutral cue-validity condition) or directed (as in the 100% or 80% cue-validity conditions), learning and transfer was observed. And so, learning was found across all conditions suggesting that, as least in this context, attention was sufficient for learning to occur. The type of transfer- feature transfer, location transfer or task transfer- may be constrained by the type of attention. Although, common mechanisms may activate both forms of attention which allow for the occurrence of feature transfer. However, other forms of transfer may depend on the type of attention. Despite inter-individual variability at initial levels of performance, all participants were at comparable levels following training. This finding suggests that attention may indeed reduce any individual differences in learning. Previous research found that individuals consistently exhibited transfer when trained with exogenous attentional cues [73].

Several factors of this study may have allowed for generalized learning to occur making it difficult to assess the effect of attention. However, this study does contribute to the growing literature that demonstrates specificity is not the only defining characteristic of PL. A complete characterization of PL also includes generalized learning and the factors involved. An increasing number of more recent papers suggest that it is unlikely that any one process or mechanism is responsible for PL. Instead, multiple components of learning likely work together to produce changes in performance [94]. Complete characterization of PL involves viewing PL as a distributed process by which degree of learning and transfer are mediated by attributes of the task and stimuli used and moderated by characteristics of the individual. By extension, the exact role of attention may vary based on the configuration of the stimulus and the task. Attention may contribute to generalized learning but the nature of the training improvements and type of transfer may well depend on the type of attention.

In summary, the present findings observed learning and feature transfer across both types of attention regardless of cue-validity. Given the time-course of the study, rapid improvements were found in a few hundred trials which may reflect an early component of the learning process. This rapid learning may partly account for the finding of learning and feature transfer across conditions. Rapid learning may be qualitatively distinct from the type of learning observed in most PL studies that employ longer training trials and sessions. Thus, it is possible that employing another task that utilizes more extensive training could result in different patterns of learning and transfer. The findings here suggest that the effect of exogenous or endogenous may depend on the speed of learning.

## References

[1] Ball K., & Sekuler R. (1981). Adaptive processing of visual motion. *J*. *Exp*. *Psychol*. *Hum*. *Percept*. *Perform*. 7, 780–794. 10.1037//0096-1523.7.4.780 6457092

[2] Sagi D. & Tanne D. (1994) Perceptual-learning–learning to see. *Current Opinion in Neurobiology*, 4(2), 195–199. 10.1016/0959-4388(94)90072-8 8038576

[3] Crist R.E., Li W, & Gilbert C.D. (2001). Learning to see: experience and attention in primary visual cortex. *Nat*. *Neurosci*.,4, 519–25. 10.1038/87470 11319561

[4] Mukai I., Kim D., Fukunaga M., Japee S., Marrett S., & Ungerleider L. G. (2007). Activations in visual and attention-related areas predict and correlate with the degree of perceptual learning. *The Journal of Neuroscience*, 27, 11401–11411. 10.1523/JNEUROSCI.3002-07.2007 17942734 PMC6673045

[5] Yotsumoto Y., Watanabe T., & Sasaki Y. (2008) Different dynamics of performance and brain activation in the time course of perceptual learning. *Neuron*, 57, 827–33. 10.1016/j.neuron.2008.02.034 18367084 PMC2735208

[6] Andersen G.J. (2012). Aging and vision: changes in function and performance from optics to perception. *Wiley Interdiscip*. *Rev*. *Cogn*. *Sci*.,3, 403–10. 10.1002/wcs.1167 22919436 PMC3424001

[7] Baker C. I., Peli E., Knouf N., & Kanwisher N. G. (2005). Reorganization of visual processing in macular degeneration. *J*. *Neurosci*., 25, 614–618. 10.1523/JNEUROSCI.3476-04.2005 15659597 PMC6725316

[8] Polat U. (2009) Making perceptual learning practical to improve visual functions. *Vision Research*, 49, 2566–2573. 10.1016/j.visres.2009.06.005 19520103

[9] Polat U., Schor C., Tong J.-L., Zomet A., Lev M., Yehezkel O. et al (2012). Training the brain to overcome the effect of aging on the human eye. *Scientific Reports*, 2, 278 10.1038/srep00278 22363834 PMC3284862

[10] Levi D.M. (2005) Perceptual learning in adults with amblyopia: A reevaluation of critical periods in human vision. *Dev Psychobiol*,46, 222–232. 10.1002/dev.20050 15772964

[11] Levi D. M., & Li R. W. (2009). Improving the performance of the amblyopic visual system. *Philosophical Transactions of the Royal Society of London*. *Series B*: *Biological Sciences*, 364 *(*1515), 399–407. 10.1098/rstb.2008.0203 19008199 PMC2674474

[12] Levi D. M., & Polat U. (1996). Neural plasticity in adults with amblyopia. *Proceedings of the National Academy of Sciences of the United States of America*, 93(13), 6830–6834. 10.1073/pnas.93.13.6830 8692904 PMC39113

[13] Polat U., Ma-Naim T., Belkin M., & Sagi D. (2004). Improving vision in adult amblyopia by perceptual learning. *Proceedings of the National Academy of Sciences of the United States of America*, 101(17), 6692–6697 10.1073/pnas.0401200101 15096608 PMC404107

[14] Ahissar M., & Hochstein S. (1997). Task difficulty and the specificity of perceptual learning. *Nature*,387, 401–6. 10.1038/387401a0 9163425

[15] Liu Z., & Weinshall D. (2000). Mechanisms of generalization in perceptual learning. *Vision Research*, 40(1), 97–109. 10.1016/s0042-6989(99)00140-6 10768045

[16] Jeter P. E., Dosher B. A., Petrov A., & Lu Z. L. (2009). Task precision at transfer determines specificity of perceptual learning. *Journal of Vision*, 9(3), 1–13. 10.1167/9.3.1 PMC4964592 19757940

[17] Wang R., Zhang J. Y., Klein S. A., Levi D. M., & Yu C. (2012). Task relevancy and demand modulate double-training enabled transfer of perceptual learning. *Vision Research*, 61, 33–38. 10.1016/j.visres.2011.07.019 21820004 PMC3227777

[18] Yotsumoto Y., Chang L. H., Ni R., Salat D., Andersen G., Watanabe T., et al (2010). Perceptual learning and changes in white matter in aged brain revealed by difusion-tensor imaging (DTI). *Journal of Vision*, 10(7), 912.

[19] Yotsumoto Y., Chang L.H., Ni R., Pierce R., Andersen G.J., Watanabe T., et al (2014). White matter in the older brain is more plastic than in the younger brain. *Nature Communications*, 5, 5504 10.1038/ncomms6504 25407566 PMC4238045

[20] Shibata K., Watanabe T., Sasaki Y. & Kawato M. (2011) Perceptual learning incepted by decoded fMRI neurofeedback without stimulus presentation. *Science*, 334(6061), 1413–1415. 10.1126/science.1212003 22158821 PMC3297423

[21] Yotsumoto Y. & Watanabe T. (2008). Defining a link between perceptual learning and Attention. *PLoS Biology*, 6(8), e221 10.1371/journal.pbio.0060221 18752357 PMC2525694

[22] Fiorentini A, Berardi N. (1980). Perceptual learning specific for orientation and spatial frequency. *Nature*,287, 43–44. 10.1038/287043a0 7412873

[23] Fiorentini A., & Berardi N. (1981) Learning in grating wave form discrimination: specificity for orientation and spatial frequency. *Vision Research*, 21, 1149–1158. 10.1016/0042-6989(81)90017-1 7314493

[24] Poggio T., Fahle M., & Edelman S. (1992) Fast perceptual learning in visual hyperacuity. *Science*. 256, 1018–1021. 10.1126/science.1589770 1589770

[25] Ball K., & Sekuler R. (1982). A specific and enduring improvement in visual motion discrimination. *Science*, 218, 697–698. 10.1126/science.7134968 7134968

[26] Ball K., & Sekuler R. (1987). Direction-specific improvement in motion discrimination. *Vision Research*,27, 953–65. 10.1016/0042-6989(87)90011-3 3660656

[27] Adini Y., Sagi D., & Tsodyks M. (2002). Context-enabled learning in the human visual system. *Nature*,415,790–93. 10.1038/415790a 11845209

[28] Yu C., Klein S. A., & Levi D. M. (2004). Perceptual learning in contrast discrimination and the (minimal) role of context. *Journal of Vision*, 4(3), 169–182 10.1167/4.3.4 15086307

[29] Hua T., Bao P., Huang C.B., Wang Z., Xu J., Zhou Y., et al (2010). Perceptual learning improves contrast sensitivity of V1 neurons in cats. *Current Biology*, 20, 887–94. 10.1016/j.cub.2010.03.066 20451388 PMC2877770

[30] Watanabe T., & Sasaki Y., (2015). Perceptual Learning: toward a comprehensive theory. Annu. Rev. Psychol. 10.1146/annurev-psych-010814-015214. PMC4286445 25251494

[31] Sagi D. (2011) Perceptual learning in vision research. *Vision Research*, 51, 1552–1566. 10.1016/j.visres.2010.10.019 20974167

[32] Jeter P. E., Dosher B. A., Liu S. H., & Lu Z. L. (2010). Specificity of perceptual learning increases with increased training. *Vision Research*, 50(19), 1928–1940. 10.1016/j.visres.2010.06.016 20624413 PMC3346951

[33] Szpiro S. F. A., & Carrasco M. (2015). Exogenous attention enables visual perceptual learning. *Psychological Science*, 27 *(*4*)*, 592 10.1177/0956797616636342 PMC4695399 26502745

[34] Liu Z. (1999). Perceptual learning in motion discrimination that generalizes across motion directions. *Proc*. *Natl*. *Acad*. *Sci*. USA, 96, 14085–87. 10.1073/pnas.96.24.14085 10570202 PMC24194

[35] Xiao L.Q., Zhang J.Y., Wang R., Klein S.A., Levi D.M., & Yu C. (2008) Complete transfer of perceptual learning across retinal locations enabled by double training. *Current biology*, 18, 1922–6. 10.1016/j.cub.2008.10.030 19062277 PMC3045109

[36] Dosher B.A., & Lu Z.L. (1998). Perceptual learning reflects external noise filtering and internal noise reduction through channel reweighting. *Proc*. *Natl*. *Acad*. *Sci*. USA, 95, 13988–93. 10.1073/pnas.95.23.13988 9811913 PMC25004

[37] Dosher B.A., & Lu Z.L. (1999). Mechanisms of perceptual learning. *Vision Research*, 39, 3197–221. 10.1016/s0042-6989(99)00059-0 10615491

[38] Liu J., Lu Z.L., & Dosher B.A. (2010). Augmented Hebbian reweighting: interactions between feedback and training accuracy in perceptual learning. *Journal of Vision*,10, 29. 10.1167/10.10.29 PMC12532228 20884494

[39] Petrov A.A., Dosher B.A., & Lu Z.L. (2005). The dynamics of perceptual learning: an incremental reweighting model. *Psychol*. *Rev*., 112, 715–43. 10.1037/0033-295X.112.4.715 16262466

[40] Dosher B.A., Jeter P., Liu J., & Lu Z.L. (2013). An integrated reweighting theory of perceptual learning. *Proc*. *Natl*. *Acad*. *Sci*. USA, 110, 13678–83. 10.1073/pnas.1312552110 23898204 PMC3746919

[41] Ahissar M., & Hochstein S. (2004). The reverse hierarchy theory of visual perceptual learning. *Trends in Cognitive Sciences*, 8, 457–464. 10.1016/j.tics.2004.08.011 15450510

[42] Aberg K. C., Tartaglia E. M., & Herzog M. H. (2009). Perceptual learning with Chevrons requires a minimal number of trials, transfers to untrained directions, but does not require sleep. *Vision Research*, 49(16), 2087–2094. 10.1016/j.visres.2009.05.020 19505495

[43] Seitz A., & Watanabe T. (2005). A unified model for perceptual learning. *Trends Cogn*. *Sci*. 9:329–34. 10.1016/j.tics.2005.05.010 15955722

[44] Watanabe T., Nanez J.E., & Sasaki Y. (2001). Perceptual learning without perception. *Nature*, 413, 844–48. 10.1038/35101601 11677607

[45] Seitz A. R., & Watanabe T. (2009). The phenomenon of task-irrelevant perceptual learning. *Vision Research*, 49, 2604–2610. 10.1016/j.visres.2009.08.003 19665471 PMC2764800

[46] Gutnisky D.A., Hansen B.J., Iliescu B.F., & Dragoi V. (2009). Attention alters visual plasticity during exposure-based learning. *Current Biology*, 19, 555–60. 10.1016/j.cub.2009.01.063 19268592

[47] Yantis S., & Jonides J. (1990). Abrupt visual onsets and selective attention: Voluntary versus automatic allocation. *Journal of Experimental Psychology*: *Human Perception and Performance*, 16, 121–134. 10.1037//0096-1523.16.1.121 2137514

[48] Yantis S., & Jonides J. (1984). Abrupt visual onsets and selective attention: evidence from visual search. *Journal of Experimental Psychology*: *Human Perception and Performance*, 10(5),601–21. 10.1037//0096-1523.10.5.601 6238122

[49] Jonides J. & Yantis S. (1988). Uniqueness of abrupt visual onset in capturing attention. *Perception & Psychophysics*, 43, 346–354. 3362663 10.3758/bf03208805

[50] Yantis S., & Hillstrom A. P. (1994). Stimulus-driven attentional capture: Evidence from equiluminant visual objects. *Journal of Experimental Psychology*: *Human Perception and Performance*, 20, 95–107. 10.1037//0096-1523.20.1.95 8133227

[51] Yantis S. (1993). Stimulus-driven attentional capture and attentional control settings. *Journal of Experimental Psychology*: *Human Perception and Performance*, 19, 676–681. 10.1037//0096-1523.19.3.676 8331320

[52] Jonides J. (1981). Voluntary versus automatic control over the mind’s eye’s movement In: Long JB.; Baddeley AD., editors. Attention & performance IX. Erlbaum; Hillsdale, NJ: 187–203.

[53] Lu Z.L., & Dosher B.A. (2000) Spatial attention: Different mechanisms for central and peripheral temporal precues?, *Journal of Experimental Psychology*: *Human Perception and Human Performance*, 26(5), 1534–1548. 10.1037//0096-1523.26.5.1534 11039483

[54] Lu Z.L., & Dosher B.A. (1998) External noise distinguishes attention mechanisms. *Vision Research*, 38,1183–1198. 10.1016/s0042-6989(97)00273-3 9666987

[55] Theeuwes J. (2010) Top-down and bottom-up control of visual selection. *Acta Psychologica*, 135(2), 77–99. 10.1016/j.actpsy.2010.02.006 20507828

[56] Cheal M., Lyon D. (1991) Central and peripheral precuing of forced-choice discrimination. *Quarterly Journal of Experimental Psychology*: *Human Experimental Psychology*. 43(4), 859–880. 10.1080/14640749108400960 1775667

[57] Nakayama K., & Mackeben M. (1989). Sustained and transient components of focal visual attention. *Vision Research*, 29(11), 1631–1647. 10.1016/0042-6989(89)90144-2 2635486

[58] Folk C. L., Remington R. W., & Johnston J. C. (1992). Involuntary covert orienting is contingent on attentional control settings. *Journal of Experimental Psychology*: *Human Perception and Performance*, 18(4), 1030–1044. 1431742

[59] Posner M. I. (1980). Orienting of attention. *Quarterly Journal of Experimental Psychology*, 32(1), 3–25. 10.1080/00335558008248231 7367577

[60] Carrasco M. (2011). Visual attention: The past 25 years. *Vision Research*, 51, 1484–1525. 10.1016/j.visres.2011.04.012 21549742 PMC3390154

[61] Suzuki S., & Cavanagh P. (1997). Focused attention distorts visual space: An attentional repulsion effect. *Journal of Experimental Psychology*: *Human Perception and Performance*, 23(2), 443–463. 10.1037//0096-1523.23.2.443 9104004

[62] Hikosaka O., Miyauchi S., & Shimojo S. (1993). Focal visual attention produces illusory temporal order and motion sensation. *Vision Research*, 33(9), 1219–1240. 10.1016/0042-6989(93)90210-n 8333171

[63] Giordano A. M., McElree B., & Carrasco M. (2009). On the automaticity and flexibility of covert attention: A speed-accuracy trade-off analysis. *Journal of Vision*, 9(3), 10–31. 10.1167/9.3.10 19757969 PMC3684150

[64] Yeshurun Y., Montagna B., & Carrasco M. (2008). On the flexibility of sustained attention and its effects on a texture segmentation task. *Vision Research*, 48(1), 80–95. 10.1016/j.visres.2007.10.015 18076966 PMC2638123

[65] Yeshurun Y., & Carrasco M. (1998). Attention improves or impairs visual performance by enhancing spatial resolution, *Nature*, 396(6706), 72–75. 10.1038/23936 9817201 PMC3825508

[66] Muller H. J., & Rabbitt P. M. (1989). Reflexive and voluntary orienting of visual attention: Time course of activation and resistance to interruption. *Journal of Experimental Psychology*: *Human Perception and Performance*, 15(2), 315–330. 10.1037//0096-1523.15.2.315 2525601

[67] Posner M.I., Nissen M.J., & Ogden W.C. (1978). Attended and unattended processing modes: The role of set for spatial location Pick J.H.I., Saltzman E. (Eds.), *Modes of perceiving and processing information* (pp.137-157). Erlbaum, Hillsdale, NJ, 137–157.

[68] Hopfinger J. B., & West V. M. (2006). Interactions between endogenous and exogenous attention on cortical visual processing. *NeuroImage*, 31(2), 774–789. 10.1016/j.neuroimage.2005.12.049 16490366

[69] Posner M.I. & Cohen Y. (1984) Components of Visual Orienting In: Bouma H. and Bouwhuis D.G., Eds., Attention and Performance X: Control of Language Processes, Erlbaum, Hillsdale, 531–556.

[70] Byers A. & Serences J.T. (2012). Exploring the relationship between perceptual learning and top-down attentional control. *Vision Research*, 74, 30–39. 10.1016/j.visres.2012.07.008 22850344 PMC3501545

[71] Tsushima Y., & Watanabe T. (2009). Roles of attention in perceptual learning from perspectives of psychophysics and animal learning. *Learn*. *Behav*., 37, 126–32. 10.3758/LB.37.2.126 19380889 PMC2866071

[72] Mukai I., Bahadur K., Kesavabhotla K., & Ungerleider L. G. (2011). Exogenous and endogenous attention during perceptual learning differentially affect post-test target thresholds. *Journal of Vision*, 11(1), Article 25. 10.1167/11.1.125 PMC3268656 21282340

[73] Donovan I., Szpiro S., & Carrasco M. (2015). Exogenous attention facilitates perceptual learning transfer within and across visual hemifields. *Journal of Vision*, 15(10), Article 11. 10.1167/15.10.11 26426818 PMC4594468

[74] Donovan I., & Carrasco M. (2018). Endogenous spatial attention during perceptual learning facilitates location transfer. *Journal of Vision*, 18(11):7,1–16. 10.1167/18.11.7 30347094 PMC6181190

[75] Reynolds J. H., & Heeger D. J. (2009). The normalization model of attention. *Neuron*, 61(2), 168–185. 10.1016/j.neuron.2009.01.002 19186161 PMC2752446

[76] Donovan I., Shen A., Barbot A., & Carrasco M. (2019, 5). Does exogenous spatial attention facilitate perceptual learning transfer in acuity and hyperacuity tasks? *Journal of Vision*, 19(10):26d 10.1167/19.10.26d.

[77] Hochstein S., & Ahissar M. (2002). View from the top: hierarchies and reverse hierarchies in the visual system. *Neuron*,36, 791–804. 10.1016/s0896-6273(02)01091-7 12467584

[78] Shibata K., Sagi D., & Watanabe T. (2014) Two stage model of perceptual learning, *the Year in Cognitive Neuroscience*, *Annual Review of New York Academy of Sciences*, 1316, 18–28. 10.1111/nyas.12419 PMC4103699 24758723

[79] Shibata K., Sasaki Y., Kawato M., & Watanabe T. (2016) Neuroimaging evidence for two types of plasticity in association with visual perceptual learning. *Cerebral Cortex*, 26 (9), 3681–3689. 10.1093/cercor/bhw176 27298301 PMC5004756

[80] Shibata K., Yamagishi N., Ishii S., & Kawato M. (2009). Boosting perceptual learning by fake feedback. *Vision Research*, 49, 2574–2585. 10.1016/j.visres.2009.06.009 19531366

[81] Peli E. (1990). Contrast in complex images. *J*. *Opt*. *Soc*. *Am*. *A*., 7(10), 2032–2040. 10.1364/josaa.7.002032 2231113

[82] Mayfrank L., Kimmig H., & Fischer B. (1987). The role of attention in the preparation of visually guided saccadic eye movements in man In O’Reganm J. K.& Levy-Schoen A. (Eds.), Eye movements: From physiology to cognition (pp. 37–45). NY: North-Holland.

[83] Watson A. A. B., & Pelli D. G. D. (1983). QUEST: A Bayesian adaptive psychometric method. *Attention*, *Perception*, *& Psychophysics*, 33, 113–120. 10.3758/bf03202828 6844102

[84] Hautus M.J. (1995). Corrections for extreme proportions and their biasing effects on estimated values of d’. *Behavior Research*, *Methods*, *Instruments & Computers*, 27, 46–51. 10.3758/BF03203619.

[85] Fahle M., & Henke-Fahle S. (1996) Interobserver variance in perceptual performance and learning. *Investigative Ophthalmology & Visual Science*, 37, 869–877. 8603871

[86] Fine I., & Jacobs R.A. (2002). Comparing perceptual learning tasks: a review. *Journal of Vision*, 2(2), 190–203. 10.1167/2.2.5 12678592

[87] Kumar T., & Glaser D. A. (1993). Initial performance, learning and observer variability for hyperacuity tasks. *Vision Research*, 33, 2287–2300. 10.1016/0042-6989(93)90106-7 8273293

[88] Fahle M., & Edelman S., (1993), Long-term learning in vernier acuity: Effects of stimulus orientation, range and of feedback. *Vision Research*, 33(3), 397–412. 10.1016/0042-6989(93)90094-d 8447110

[89] Karni A., & Sagi D. (1993). The time course of learning a visual skill. *Nature*,365, 250–52. 10.1038/365250a0 8371779

[90] Zhang T., Xiao L. Q., Klein S. A., Levi D. M., & Yu C. (2010). Decoupling location specificity from perceptual learning of orientation discrimination. *Vision Research*, 50(4), 368–374, 10.1016/j.visres.2009.08.024 19716377

[91] Zhang J. Y. & Yang Y. X. (2014). Perception learning of motion direction discrimination transfers to an opposite direction with TPE training. *Vision Research*, 99, 93–98. 10.1016/j.visres.2013.10.011 24184566

[92] Wang R., Zhang J. Y., Klein S. A., Levi D. M., Yu C. (2014). Vernier perceptual learning transfers to completely untrained retinal locations after double training: A “piggybacking” effect. *Journal of Vision*, 14 (13): 12, 1–12, 10.1167/14.13.12 25398974 PMC4233766

[93] Sireteanu R., & Rettenbach R. (2000). Perceptual learning in visual search generalizes over tasks, locations, and eyes. *Vision Research*, 40(21), 2925–2949. 10.1016/s0042-6989(00)00145-0 11000393

[94] Maniglia & Seitz (2017), "Towards a whole brain model of Perceptual Learning", *Current Opinion in Behavioral Sciences*, 20, 47–55. 10.1016/j.cobeha.2017.10.004 29457054 PMC5810967

